# The role of phages in plant-associated microbial communities

**DOI:** 10.1042/EBC20250035

**Published:** 2026-09-02

**Authors:** Renée E. Smith, Dominique Holtappels

**Affiliations:** School of Integrative Plant Science, Plant Pathology and Plant-Microbe Biology Section, Cornell University, Cornell AgriTech, Geneva, NY, U.S.A.

**Keywords:** Bacteriophage, Microbiome, Microbiome function, Virome

## Abstract

Microbial communities deliver essential functions in ecosystems. In plant environments, the plant microbiome facilitates nutrient uptake, supports plants during abiotic stress, and counteracts disease. As implementation of synthetic microbial communities becomes more of a realistic strategy for mitigating the effects of biotic and abiotic stressors on plant productivity, it is increasingly important to understand how interactions between microbes, which are essential for ecosystem function (hub microbes), are maintained. Recent research highlights the ecological role of bacteriophages, the viruses of bacteria, in host-associated microbial communities. Current evidence demonstrates the influence of the phageome on microbiomes, ranging from effects on an individual (transduction, lysogenic conversion, and evolutionary pressure) to entire populations and communities, such as Kill-the-Winner dynamics. These dynamics appear to affect the overall function of microbial communities and support plant growth. In this review, we lay out recent insights on the role of bacteriophages in plant-associated microbiomes through an eco-evolutionary lens and future directions of research to broaden our understanding of the ecological implications of bacteriophages.

## The role of microbiomes to support plant health

Microorganisms are integral for facilitating ecosystem function and services [1,2]. Microbial community composition and diversity can predict ecosystem multifunctionality such as carbon use efficiency, denitrification, micronutrient efficiency, and plant productivity [3,4]. The overall resilience of an ecosystem, or an individual host, is impacted by the microbial diversity and makeup. Resilience at both the host and ecosystem levels can be defined as the rate at which a system can either be returned to the original state or to another alternative stable state [5]. In non-host systems, such as in the soil, bacterial biomass, diversity, and richness have an inconsistent response to environmental stress such as drought or heat [6]. However, more diverse soil microbial communities can increase stress resilience through redundancy, complementarity, and the maintenance of key functional groups in essential ecosystem processes, allowing a faster recovery following an abiotic stress (temperature, humidity, salinity, pH, among other non-living environmental factors) [5,7]. Diverse microbial communities serve as an ecological buffer in the exposure to both abiotic and biotic stressors.

Plant microbiomes are host-associated microbial communities that include endophytes living at least part of their life cycle within the plant and epiphytes that live on the surface of plants. Epiphytes include microbes living in the phyllosphere (above-ground surfaces of the plant such as the leaves, stems, flowers, and fruits) and/or rhizosphere (the below-ground parts of the plant such as roots) [8–10]. Diverse communities are crucial in the plant environment for supporting plants during abiotic stress, counteracting plant disease, and improving plant nutrition and growth [11–15]. Efforts have been made toward making stable and diverse synthetic microbial communities (SynComs) to improve crop resilience and agricultural yield [16–18]. Building on these ideas, recent research efforts are focusing on identifying highly interactive members of the core microbiota known as “hub microbes” to create longer-lasting, customized SynComs to improve plant health [14,19–21]. In host-associated microbiomes, the interactions between the members of the microbial community and their environment can serve as a more accurate way to assess functionality at an individual and ecosystem level [22–24]. Therefore, researching and reporting on additional factors that can influence the interactions between microbes and hosts can inform the overall function of the system.

One of these factors influencing microbial interactions is bacteriophages or phages, the viruses of bacteria, which are ubiquitously found across all ecosystems on earth [25–29]. Phages fundamentally alter how microbes respond as an individual, within a population, and among a larger community [30–32]. These viruses exhibit either lytic, pseudolysogenic, or lysogenic lifestyles, which have implications for the ecological dynamics they facilitate. Lytic phages infiltrate the bacterial host cell, manipulate cellular machinery to synthesize new phage genetic information and assemble new phages before lysing the bacterial cell. Upon infection, lytic phages do not always immediately take over the host’s cellular machinery. Phages have been described to engage in pseudolysogeny, a state in which the phage resides inside the bacterial cell after infection, without integrating into the host genome, and remains “dormant” until specific favorable conditions are met [33,34]. In contrast, temperate phages that undergo lysogeny infiltrate a host cell and insert their own genome into the bacterial genome, becoming a prophage, and replicate with the host. By inserting directly into the genome of their host, these temperate phages can increase genetic diversity through facilitating horizontal gene transfer (HGT), as they have the ability to transfer genetic cargo from one host to the next, a process referred to as lysogenic conversion [35]. Through the different phage lifestyles, phages have direct interactions with their host bacteria in terms of their abundance and evolutionary trajectory, exerting selective pressure, facilitating HGT, and triggering phage defense systems.

In this mini-review, we will discuss current knowledge on the effects of bacteriophages on individual hosts, host populations, and the wider community, specifically focusing on plant-associated microbiomes. Additionally, we will explore how phages influence microbial adaptation through the dispersal of genetic material in microbial communities and evolutionary pressure. Finally, we will lay out how phages alter the microbiome function and highlight future areas of research for phage impact on ecological function in plant host-associated systems.

## Effects of phages on individual hosts and local adaptation in the plant environment

**Figure 1:**
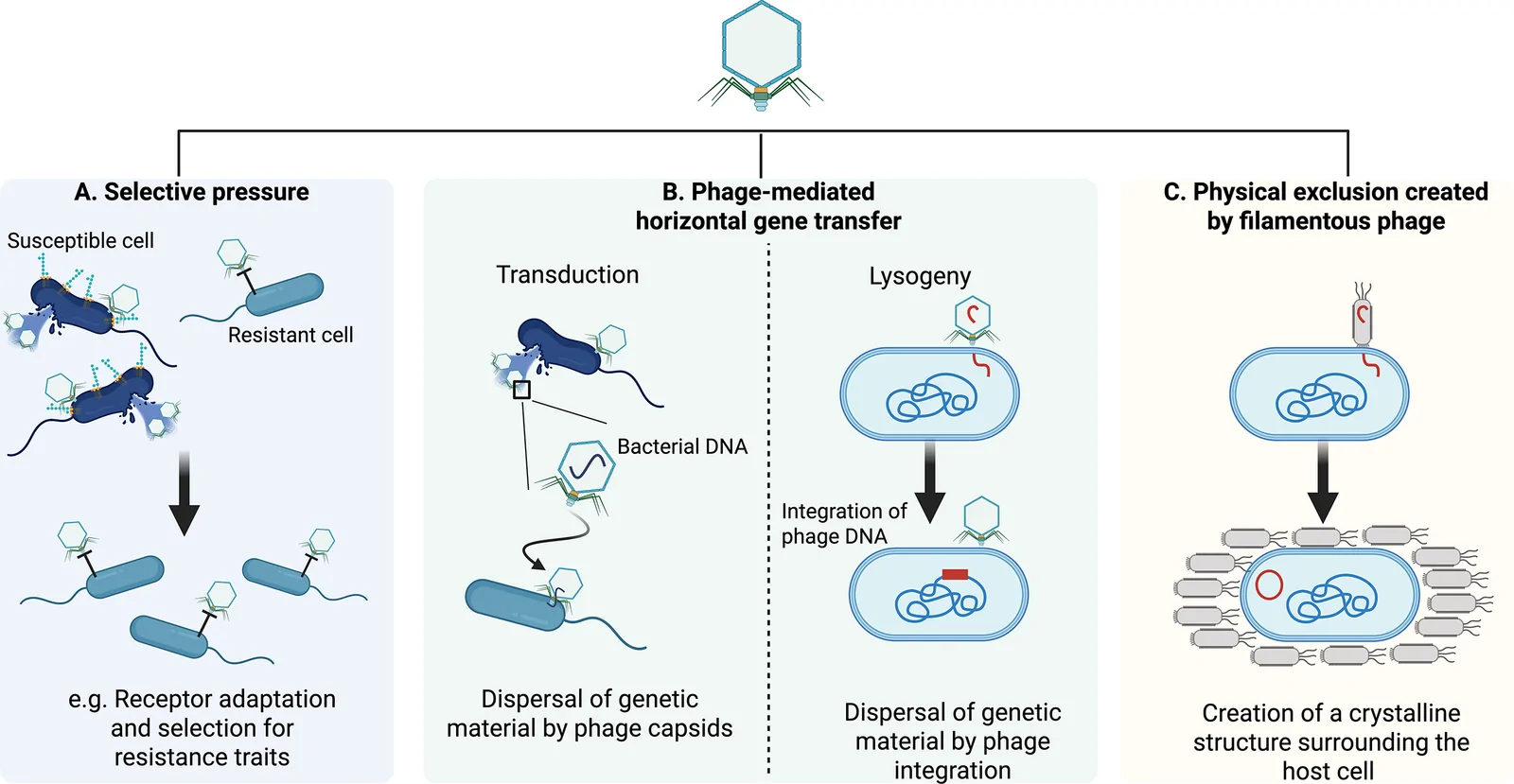
Bacteriophages can modulate the evolution and ecology of their hosts (**A**) Lytic phages directly lyse their bacterial host upon infection, creating a strong selective pressure for resistant clones in the population (light blue bacteria) by selecting for altered phage receptors and other resistance traits. (**B**) Phage-mediated HGT (including transduction and lysogeny) facilitates the dispersal of genetic cargo such as antibiotic resistance genes, auxiliary metabolic genes, phage resistance mechanisms, and virulence genes. In case of transduction, phages package bacterial DNA (depicted in blue) in their capsids, which can be distributed to other, susceptible bacteria within the community. Temperate phages, on the other hand, have the ability to integrate directly into the bacterial genome and act as self-regulating mobile genetic elements (phage DNA depicted in red). (**C**) Filamentous phages form crystalline structures around the host cell, providing a physical barrier for other phages and bactericidal compounds.

Phages influence the evolution of their bacterial hosts in different ways, ranging from selective pressure imposed by lytic phages to phage-mediated HGT through transduction and lysogenic conversion, as well as changing physical properties of a bacterium by creating crystalline structures around the bacterial cell (Figure 1).

In laboratory conditions, the evolutionary pressure imposed by lytic phages has repeatedly been associated with adaptation of phage receptors. Numerous examples of receptor analyses and coevolution studies highlight the cost of resistance as a result of receptor adaptations, primarily at the level of LPS (in the case of Gram-negative bacteria) and flagella [36–44]. While some of these examples highlight the trade-off effects associated with phage resistance in plant pathogenic bacteria such as *Xanthomonas campestris, Pseudomonas syringae*, and *Agrobacterium* spp. among others, there is a critical need to understand these dynamics in host-associated environments such as the plant directly, as reviewed elsewhere [45].

Besides direct evolutionary pressure, certain groups of phages have the ability to directly disperse genetic information. Phage-mediated HGT makes phageomes potential reservoirs for fitness-related genes such as virulence, phage defense, auxiliary metabolic, and antibiotic resistance genes (ARGs) [46–49]. Transduction, including specialized, lateral, and generalized transduction, is the phenomenon by which phages package bacterial DNA in their capsids and disperse genes from one cell to the next. Specialized transduction occurs when a prophage excises additional DNA from the host genome when the prophage enters the lytic stage, leading to the packaging of genes directly adjacent to the prophage into the capsid. Lateral transduction, the most recently characterized form of transduction, has proven to be a more efficient way of mobilizing bacterial chromosomes than other mobile genetic elements [50]. This process takes place when prophage excision is postponed following initial activation. Rather than immediate excision (as it occurs in specialized transduction), the prophage undergoes *in situ* replication, amplifying its genetic copies. Consequently, when the prophage eventually excises and packages DNA into the phage capsid, it incorporates significantly more kilobases of genetic material and frequently encompassing extensive regions of neighboring bacterial genes, or sometimes even only bacterial DNA [50–52]. In contrast with specialized and lateral transduction, generalized transduction can occur when lytic phages, such as members from the Viunalikevirus genes, including LIMEstone1 and LIMEstone2, infecting the plant pathogen *Dickeya solani*, inadvertently pack random genetic information from anywhere in the bacterial host chromosome [53]. Through these mechanisms, phages can act as vehicles to disperse genetic material from one susceptible cell to the next within the microbial community and likely contribute to local adaptation.

Besides the random packaging and dispersal of bacterial DNA through transduction, temperate phages have been reported to carry genetic cargo in their genomes. These temperate phages are in essence self-replicating mobile genetic elements with high fitness trade-offs to their hosts [54] and follow the Mutualism-Parasitism Continuum hypothesis: prophages embedded in bacterial genomes have been described to carry genes of interest to the bacterial host while having the ability to lyse the host upon specific environmental triggers. These beneficial genes, including virulence genes, phage defense systems, and auxiliary metabolic genes (AMGs), among others, blur the ecological line between parasitism and mutualism in phage-bacteria relationships, as the integration and continued replication of a prophage can be essential for bacterial survival under certain conditions and environments [55,56]. The transfer of these fitness-related genes is hypothesized to be evolutionarily advantageous toward the continuous replication of the phage [57]. The classic examples of prophages carrying virulence genes and engaging in lysogenic conversion are prophage-encoded genes for the Shiga toxin in *Escherichia coli* and the cholera toxin in *Vibrio cholerae* [58]. In the case of plant pathogens, Hulin et al. showed through genome mining efforts that prophages carry effector genes in *P. syringae*. They further demonstrated that these prophages disperse the effector protein HopAR1 from a pathogenic isolate of *Pseudomonas syringae* into another naïve isolate while co-inoculated on the leaf surface [59]. This suggests that *P. syringae* prophages indeed contribute to the dispersal of effector genes and potentially alter pathogen-plant interactions. In another study, the presence of a reactive oxygen species-scavenging peroxidase in prophage SC2 was identified, embedded in the genome of *Candidatus Liberibacter* asiaticus, causal agent of Huanglongbing disease, or citrus greening, in citrus [46]. Even though temperate phages have the potential to distribute virulence factors within the microbial community, phage presence has more often been reported to be a decrease of the pathogenicity of bacterial plant pathogens [47–49, 60].

Besides virulence genes, other genes conferring a benefit to the host have been found in temperate phages such as phage defense mechanisms. These mechanisms are hypothesized to play a role in superinfection exclusion by preventing or eliminating other phages from infecting the same cell [61]. An example discovered in 2025 in *Salmonella enterica* includes a prophage-encoded abortive infection system composed of RemS and PokE. While the former is an ATPase, the latter is a pinholin-like element that triggers membrane depolarization upon superinfection [62]. Additionally, self-immunity elements can be encoded within the integrated phage, granting lysogens (prophage-infected bacteria) autoimmunity and ensuring the continuation of phage replication [63]. In the plant microbiome specifically, prophages integrated in the plant pathogenic host *Erwinia amylovora* contained a gene involved with the CBASS immune recognition and a gene for one of the proteins involved in the Gabija anti-phage defense complex [64,65]. Interestingly, the same phage with a CBASS gene also contained genes to disarm restriction modification (anti-RM) and CRISPR–Cas systems (ACR), making it able to disrupt phage defense systems and increase defense of the bacterial host from other lytic phages [64]. These examples suggest that temperate phages potentially impact bacterium-virus interactions, yet detailed studies are necessary to evaluate the effectiveness of these (occasionally partial) mechanisms to effectively defend against superinfecting phages. The presence of these mechanisms, however, can also impose an evolutionary trade-off for the bacterial host: mechanisms such as CRISPR–Cas and restriction enzymes not only degrade phage nucleic acids within the cell, but they also inhibit the entrance of other foreign DNA such as plasmids and transposons and thus restrict HGT and the ability of the bacterial host to adapt to its environment [66].

The impact of temperate phages on bacterial physiology and metabolism is further underlined by another type of fitness-associated trait found within prophages, including AMGs. AMGs are genes acquired by a temperate phage from a previous host that improve bacterial metabolism through increased nutrient acquisition or usage efficiency to improve viral replication [67]. In compost systems, a positive correlation between nutrient turnover and phage-host ratio was described related to AMG transfer from both lytic (through generalized transduction) and temperate phages (through integration [68]). By increasing AMG dispersion, an increased diversity of phages can be correlated with improved soil remediation from pollutants and pesticides, enabling it to become arable land quicker [69–71]. From the pathogenic perspective, a 2024 study found, for example, that soils surrounding apricot trees contained *Pseudomonas syringae-*associated phages with predicted AMGs encoding proteins involving nucleic acid synthesis, cellular metabolism, and stress tolerance, such as tellurite resistance [72]. These insights provide evidence that prophages potentially increase the fitness of plant pathogens in agricultural settings. More research is needed to identify the effects of these phages on pathogen evolution and persistence. However, the presence of these genes is often identified using computational approaches, raising the question whether these genes indeed confer a benefit to the bacterial host. As such, the role of viral AMGs has been discussed in recent years, and caution should be taken not to over-interpret the presence of specific genes in viral contigs and genomes [73].

A final class of genes found in temperate and prophages are ARGs and toxin production or defense, in which phages alter the fitness of their host, particularly in competitive environments. The potential for phages to transfer ARGs is a major concern in medical environments and is well documented in wastewater and soils [74,75]. This has been described as a barrier for utilizing bacteriophages as a biological, antibiotic-free control for human pathogens. However, a study investigating phages from hospital wastewater systems found that only 0.14% of viral contigs contained ARGs [76]. In the plant microbiome, there are unique challenges for managing the spread of antibiotic resistance across microbial communities, such as seedborne and soil-borne bacteria as vectors for ARGs in endophytic bacteria [77,78]. While no studies to date have confirmed phage-mediated spread of ARGs to plant pathogens, there are several examples of phages containing ARGs in the rhizome and phyllosphere in nonpathogenic bacteria [79–81].

While phages can transfer genes related to antibiotic resistance, they also are implicated in the transfer of toxin production genes, which kill off other competitive bacteria. Laboratory strains of phage in *Bacillus subtilis*, a bacterium found in the human gut, soil, and plant microbiome, contained a biosynthetic gene cluster (BGC, or phage-encoded pBGC), which encoded the S-linked glycopeptide sublancin [82,83]. Additionally, *Pectobacteria carotovorum*-associated phages contained a pBGC for carocin [82,84]. Both sublancin and carocin are bacteriocins, a type of antibiotic with a small and specific host range. These examples suggest that temperate phages may play a role in directing microbe–microbe interactions and increase the competitive advantage of their host in competitive environments.

Another method for how phages shape the ecology of their host is demonstrated by the crystalline structure created by filamentous phages. Unlike tailed bacteriophages, these phages do not lyse their host upon infection; rather, they bud out of the cell, keeping the host alive. As these phages are excreted from the bacterial cell, they can form crystalline structures around the bacterial cell [85]. This physical barrier changes the structure of biofilms and provides the bacterium with a protective layer against other phages and bactericidal compounds such as antibiotics [86]. In addition to the physical barrier they create, filamentous phages were found both to increase and decrease host virulence and overall fitness in the tomato pathogen *Ralstonia solanacearum* [87–89]. The filamentous phage ΦRSS1 increases the virulence of *R. solanacearum* by elevating bacterial motility and up-regulating the expression of *pchA*, a global virulence regulator in *Ralstonia* [90,91]. In contrast, ΦRS551 and ΦRSM lower the expression of several pathogenicity-related genes in combination with a reduction in motility [88,91]. The latter phenomenon has also been described in *E. amylovora*, where PEar1, PEar2, PEar4, and PEar5 reduced the swimming behavior of its host [92].

## The role of phages on population dynamics and microbial diversity

Plant microbiomes are diverse arenas that dynamically change with the microbial communities, plant fitness, and abiotic environment. Phages alter the dynamics between host bacteria and surrounding communities, how the host bacteria interact with the host plant, and the interaction between the environment and the bacterial host. Several frameworks are commonly employed to understand how phages impact the diversity and ecology of nonhost microbes. Multiple phage biocontrol studies have evaluated the effects of therapeutic phages on the overall plant microbiome, ranging from phyllosphere to rhizosphere effects. These studies highlight either the absence of overall effects on microbial alpha and beta diversity after phage applications [93,94] or positive effects on microbial community diversity metrics by reducing pathogen burden [95–97]. The positive influence of phages on microbiomes can in part be explained by Kill-the-Winner (KtW) dynamics. KtW proposes that microbes within a community are able to coexist by preventing one dominant member of the community (the winner) from outcompeting others through frequency-dependent predation (see reviewed here [98]). Phages as host-specific predators disproportionally target the most abundant prey, allowing less abundant species to survive and thrive and maintaining ecosystem diversity [99]. KtW fundamentally increases community diversity by decreasing the favorability of faster-growing bacteria [100]. While previous validation for this hypothesis was either conducted in non-host-associated marine environments or with *in vitro* experiments [100,101], a 2022 study potentially found evidence of KtW dynamics in the rhizosphere of *Brassica napus* by identifying that active vOTU prevalence was positively associated with bacterial community diversity [102].

The Piggyback-the-Winner (PtW) hypothesis is more commonly used to describe the ecological dynamics between phages and the microbial community under poorer nutrient conditions with higher levels of lysogeny [103]. The PtW hypothesis states that under less ideal, nutrient-poor conditions, combined with lower multiplicity of infection, a phage is most successful when integrated as a prophage, which provides a lysogenic advantage by increasing host fitness [104,105]. Both simulated experiments and metagenomic approaches have been used to support this model in non-host-associated microbiomes [106–109]. While this framework has been used in other host-associated microbiomes, such as the human gut, it has not specifically been used and tested in plant microbiomes [110].

## Toward the ecosystem function of viromes in plant environments

**Figure 2:**
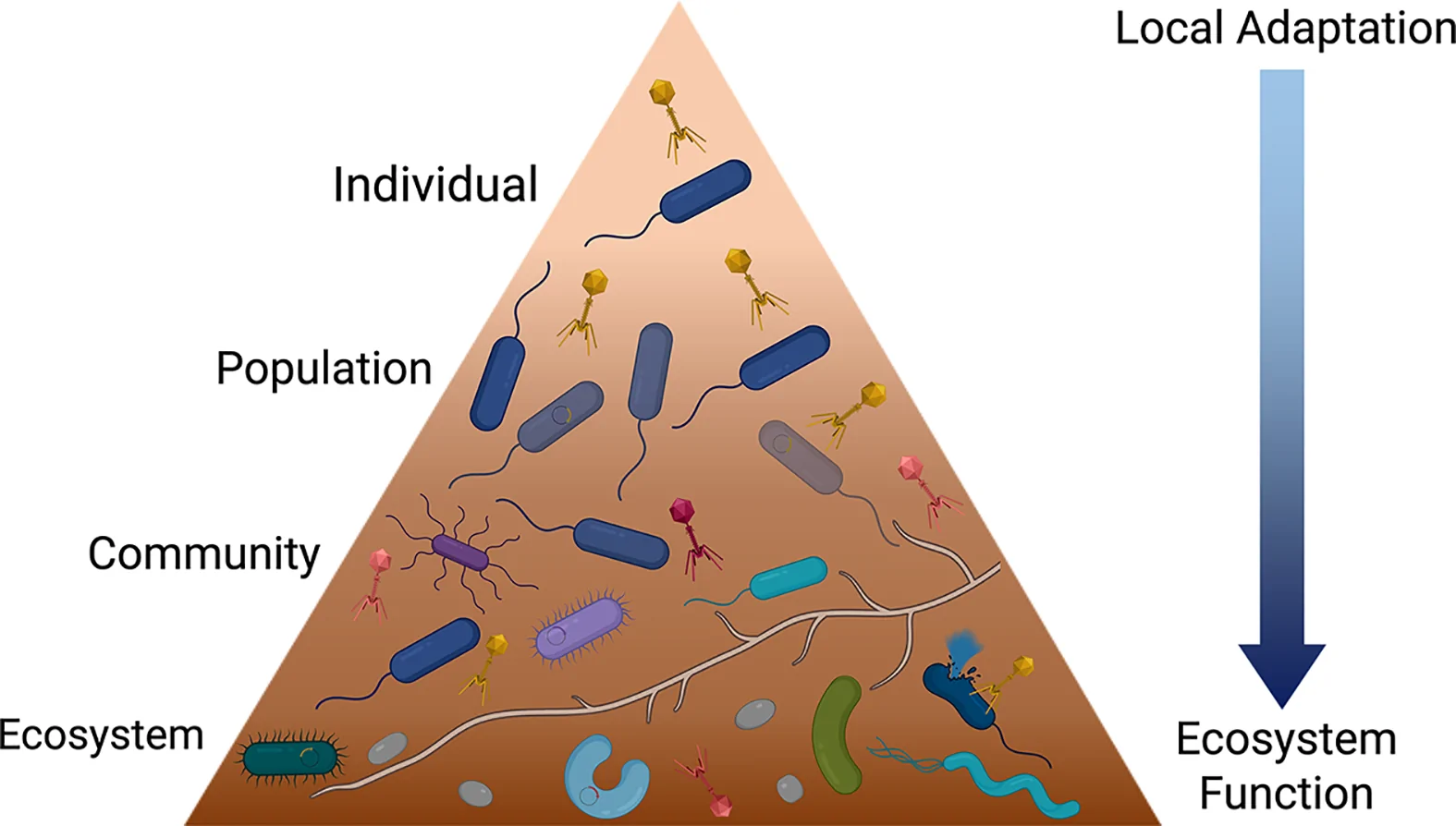
Hierarchical framework of bacteriophage effects in plant-associated microbiomes Phages impact microbial communities from individual interactions to ecosystem-level functions. At the individual level (top, individual host represented by the dark blue color), phages infect single bacterial hosts, resulting in co-evolutionary arms races and the dispersal of genetic information through phage-mediated HGT. At the population level (closely related host bacteria as represented by various blue shades), phages and their bacterial hosts are intertwined with predator-prey dynamics and KtW dynamics, which support microbiome diversity and community structure effects. By influencing bacteria at the individual and population levels, phageomes are hypothesized to play important roles at the ecosystem level (including distantly related non-host bacteria, as represented by different colors and morphologies) in both function and resilience (Figure made using BioRender).

The dynamics between phage, its primary host, other community bacteria, and the plant have larger implications for function on an ecosystem scale (Figure 2). Cell lysis as a result of lytic phage, for example, has been described to release nutrients directly into the environment and have a beneficial impact on plants [111]. Similarly, an increased diversity of phages is correlated with an increase in AMGs, which alter nutrient uptake, carbohydrate, lipid, and amino acid metabolism and the nutrients available in the environment [112]. In the soil, phages could impact soil qualities, which can relate to higher plant functions. For example, one strategy that bacteria employ to avoid phages is to create a sessile biofilm comprised of exopolysaccharides, extracellular deoxyribonucleic acids, and proteins, in combination called extracellular polymeric substances (EPS) [113]. Increased EPS in the soil is correlated with higher soil aggregation and water retention, leading to increased soil health and overall support to the plants growing in it [114]. A 2024 study was able to demonstrate the potential for phage cocktails to alter the microbiome of degraded soils by using KtW dynamics to decrease the population of dominant bacterial populations in mismanaged systems [115]. While the present study was unable to demonstrate that the addition of phage makes the community composition closer to a healthy reference soil, the soil bacteria populations did significantly shift in comparison with untreated or sterile water-treated groups. In another non-host system, paddy soils, a system that is flooded and drained regularly to grow rice, phages increased bacterial anabolism and elevated expression of carbon-fixing AMGs, which has important implications for global carbon sequestration in inundated ecosystems [116]. In addition to altering nutrient cycling and soil properties, the plant holobiome, a combination of the microbiome and virome, serves to promote plant growth, enhance seed germination, and improve disease resistance through either the disease-protective effect or induced systemic resistance [117–119]. A 2024 study found that the application of a phage cocktail made up of four lytic phages decreased the density of *R. solanacearum* and increased the native microbiome diversity. A high-frequency application of the phage cocktail also increased the relative abundance of Actinobacteria, which had pathogen-suppressive properties, acting as a secondary line of defense against successful pathogen infection [120]. The combination of beneficial phages and microbes is further illustrated by an example to reduce the impact of *R. solanacearum* on potato tubers. In the present study, phages and a PGPR *Pseudomonas lalkuanensis* A101 were combined in a microenvironment and significantly improved germination, root length, and seedling vigor [121]. By triggering increased antioxidant enzyme activity in tea plants, phage application improved resistance to *P. syringae*, which causes bud blight, without negatively impacting other plant functions [122]. Through increasing fitness of surrounding bacteria, inducing a plant immune response, or directly targeting a pathogen, phage can alter the severity of a pathogen and change plant ecosystem function.

A recent meta-analysis of 74 virome studies, predominantly from human and murine systems, investigated the relationship between virome and microbiome diversity metrics during dysbiosis [123]. The analysis revealed that virome community composition shifted in most studies in response to dysbiotic conditions, with certain viral taxa becoming disproportionately abundant. Concurrently, the normally positive correlation between virome and microbiome community richness deteriorated under dysbiotic states. These findings suggest that phages may serve as predictive indicators of microbiome health, analogous to how parasite communities can indicate broader ecosystem health status. Future research endeavors should evaluate whether this finding holds up in non-mammalian systems such as the plant environment.

## Conclusion and future perspectives

Phages, and to a broader extent, the phageome, play a role in the function of a plant microbial ecosystem through direct changes in fitness of microbial individuals, how the individuals coexist within their community, and the plant response to shifts in the microbiome. Although there has been an increase in agricultural studies looking at phageomes and viromes in plant environments, significant knowledge gaps remain. There is a need to move beyond laboratory-based phage-bacterium interaction studies toward comprehensive field-based studies that capture the complexity of natural plant environments, including temporal dynamics, spatial heterogeneity, and multi-species interactions. Future studies should prioritize developing standard methodologies for virome data analysis to enable comprehensive cross-study comparisons and meta-analyses. The integration of advanced omics approaches, including meta-transcriptomics, metabolomics, and proteomics, alongside traditional ecological methods will be essential for elucidating the mechanistic links between phage activity and ecosystem-level functions such as nutrient cycling, soil health, and overall plant productivity. Additionally, with increasing recognition of pseudolysogeny as a common phage infection strategy [124], the role of environmental factors in shaping phage-bacterium dynamics and outcomes across different agricultural and natural systems should be investigated. Finally, the development of predictive models that incorporate phage dynamics into existing microbiome frameworks could revolutionize our ability to design targeted interventions for sustainable and regenerative farming practices and ecosystem management. As we advance toward precision microbiome engineering, understanding how phageomes contribute to plant holobiome resilience and function will be fundamental for addressing global challenges in food security and environmental sustainability.

## Summary

- Diverse microbial communities support plants during stress, prevent disease, and improve growth, with efforts underway to create SynComs to enhance crop resilience.
- Phages alter bacterial evolution through selective pressure, facilitate HGT, and can either increase or decrease bacterial pathogenicity depending on the context.
- Through mechanisms such as KtW dynamics, phages help maintain microbiome diversity by preventing dominant species from outcompeting others, while biocontrol studies show phages can either maintain or positively influence overall microbiome diversity.
- The plant holobiome (microbiome + virome) promotes plant growth, enhances disease resistance, improves soil health through nutrient cycling, and may serve as predictive indicators of microbiome health, though more field-based research is necessary to fully understand these complex interactions and their outcomes.

## Open Access

Open access for this article is provided via our Subscribe to Open programme, made possible through the support of our subscribing institutions. Learn more here: https://portlandpress.com/pages/open_access_options_and_prices#conditional.

## Abbreviations

AMGs: auxiliary metabolic genes
ARGs: antibiotic resistance genes
BGC: biosynthetic gene cluster
EPS: extracellular polymeric substances
HGT: horizontal gene transfer
KtW: Kill-the-Winner
PtW: Piggyback-the-Winner
SynComs: synthetic microbial communities
